# Grasping of Real-World Objects Is Not Biased by Ensemble Perception

**DOI:** 10.3389/fpsyg.2021.597691

**Published:** 2021-04-12

**Authors:** Annabel Wing-Yan Fan, Lin Lawrence Guo, Adam Frost, Robert L. Whitwell, Matthias Niemeier, Jonathan S. Cant

**Affiliations:** ^1^Department of Psychology, University of Toronto Scarborough, Toronto, ON, Canada; ^2^The Department of Psychology, The University of British Columbia, Vancouver, BC, Canada

**Keywords:** ensemble perception, grasping, perception, electromyography, support vector machine classification, two visual stream hypothesis, action perception dissociation

## Abstract

The visual system is known to extract summary representations of visually similar objects which bias the perception of individual objects toward the ensemble average. Although vision plays a large role in guiding action, less is known about whether ensemble representation is informative for action. Motor behavior is tuned to the veridical dimensions of objects and generally considered resistant to perceptual biases. However, when the relevant grasp dimension is not available or is unconstrained, ensemble perception may be informative to behavior by providing gist information about surrounding objects. In the present study, we examined if summary representations of a surrounding ensemble display influenced grip aperture and orientation when participants reached-to-grasp a central circular target which had an explicit size but importantly no explicit orientation that the visuomotor system could selectively attend to. Maximum grip aperture and grip orientation were not biased by ensemble statistics during grasping, although participants were able to perceive and provide manual estimations of the average size and orientation of the ensemble display. Support vector machine classification of ensemble statistics achieved above-chance classification accuracy when trained on kinematic and electromyography data of the perceptual but not grasping conditions, supporting our univariate findings. These results suggest that even along unconstrained grasping dimensions, visually-guided behaviors toward real-world objects are not biased by ensemble processing.

## Introduction

Ensemble perception refers to the ability of the visual system to extract summary representations of groups of similar objects (ensembles) across various visual domains. For example, observers can accurately report the mean size of an array of different-sized circles, while, paradoxically, providing poor estimates of the size of the individual circles that are biased toward the mean size of the set (Ariely, 2001). This observation has been replicated (Chong and Treisman, 2003; Brady and Alvarez, 2011) and extended to other domains such as spatial position (Alvarez and Oliva, 2008) and orientation (Dakin and Watt, 1997; Parkes et al., 2001), and can be extracted across multiple visual domains in parallel (Emmanouil and Treisman, 2008; Attarha and Moore, 2015; Yörük and Boduroglu, 2020). Ensemble summary statistics are thought to provide the visual system with a means to make computational simplifications, which translates into lower requirements for the storage of visual information, and the preservation of a coherent percept *via* the reduction of artifactual noise that can arise from the loss of spatial resolution with increased eccentricity. In-line with this viewpoint, ensemble perception is refractory to conditions of reduced or dispersed attention (Chong and Treisman, 2005; Alvarez and Oliva, 2008, 2009; Chen et al., 2020), providing “gist” information that can guide subsequent attentional shifts (see review by Alvarez, 2011). Ensemble perception has been shown to extend beyond the mean to other statistics such as the variance and range of a set of items (Ariely, 2001; Utochkin and Vostrikov, 2017; Khayat and Hochstein, 2018; Sama et al., 2021). Importantly, these statistics (particularly variance and range), can be useful in aiding the detection of outliers and may also be integral to visual search mechanisms (Ariely, 2001). Critically, while there is an abundance of research investigating the nature of ensemble processing in the perceptual domain, very little research has focused on understanding how and if ensemble processing informs object-directed action.

Vision is the dominant sense used to guide action in everyday life. Reaching out to pick up a goal object is an intuitively trivial action performed routinely in daily life. These intuitions mask a complex process that must consider not only the agent’s intention but also the target’s 3D geometry, material properties, and spatial relationships with our eyes, body, and limbs. Motion capturing techniques have shown, for decades, that the hand, wrist, and fingers smoothly preconfigure in-flight to suit the spatial and functional features of the target (Jeannerod, 1981, 1984, 1988; Stelmach et al., 1994; Cuijpers et al., 2004; Whitwell et al., 2008). For example, the hand’s in-flight aperture (grip aperture) scales to the size of the goal object while the wrist rotates smoothly to suit the orientation of the object as the reach unfolds (e.g., Jeannerod, 1988; Kelso et al., 1994; for review, see Jeannerod, 1999). A number of different lines of evidence strongly suggest that the visual-guidance of actions like reaching out to pick up a goal object, is under the control of visuomotor modules housed in the intraparietal cortical areas of the “dorsal stream” (for review, see Milner and Goodale, 2006; Kravitz et al., 2011). In non-human primates, support for this proposal stems from single- (e.g., Taira et al., 1990; Murata et al., 2000) and multi-unit recordings (e.g., Schaffelhofer and Scherberger, 2016), cortical cooling (e.g., Gallese et al., 1994; Kermadi et al., 1997) and lesion experiments (e.g., Battaglini et al., 2002); while in humans, support comes from neuroimaging (e.g., Culham et al., 2003; Frey et al., 2005), transcranial magnetic stimulation (e.g., Tunik et al., 2005; Rice et al., 2007; Le et al., 2014, 2017), and neuropsychological work (e.g., Goodale et al., 1994; Whitwell et al., 2020).

One of the most counterintuitive findings to come out of the kinematic study of reaching and grasping is that the hand’s in-flight grip aperture is relatively refractory to the perceptual distortions of size induced by pictorial illusions. For example, in the Ebbinghaus illusion, two identically sized circles appear larger or smaller when they are surrounded by an annulus of smaller or larger circles, respectively. Aglioti et al. (1995) replaced the 2D inner disks, which are subjected to the illusion, with graspable 3D disks that were just as susceptible to the illusion when viewed face-on. When participants were asked to choose to pick up one or the other disk based on a same/different judgment about their relative size, Aglioti et al. (1995) found that although their choice was based almost exclusively on the illusory size, grip aperture remained tuned to the real size of the disk, resisting the influence of the perceived size of the illusion. This dissociation was later replicated (Haffenden and Goodale, 1998; Marotta et al., 1998; de Grave et al., 2005), and subsequent studies have extended it to other illusions (e.g., Brenner and Smeets, 1996; Servos et al., 2000; Bartelt and Darling, 2002; Ganel et al., 2008; Stöttinger et al., 2012; Whitwell et al., 2016, 2018; Smeets et al., 2020; though see Kopiske et al., 2016; Whitwell and Goodale, 2017; and Smeets and Brenner, 2006 for review). Furthermore, grip aperture is refractory to attentional crowding, which reduces the sensitivity to the size of targets embedded in a cluster of distractor objects (Chen et al., 2015). Thus, ensemble perception reduces its fidelity to individual target features like target size in favor of group statistical analogs, whereas the visuomotor system conserves fidelity to target size. Additional compelling evidence favoring a functional and anatomical distinction between the visual analysis of object geometry for perception and action comes from cases of action blindsight and visual agnosia, in which the patient, due to their compromised visual perception, cannot reliably report the size or shape of the goal object yet, remarkably, they reliably and seamlessly exploit these spatial features to inform the movements of their hand when reaching out to pick up these same objects (Goodale et al., 1991; Perenin and Rossetti, 1996; Jackson, 1999; Karnath et al., 2009; Whitwell et al., 2011, 2020). These studies strongly suggest that the visuomotor system parameterizes the details of goal-directed grasps and filters out sources of information that are typically used to inform ensemble perception.

Since the spatial organization of items in an ensemble display is typically random, these displays lack drastic size-contrast cues and the structured organization of elements seen in typical visual illusions (e.g., the Ebbinghaus illusion). Yet ensemble displays still impart a strong perceptual bias, in that the perception of a feature value of a single item from the set is routinely pulled toward the average value of the set (Brady and Alvarez, 2011; Sama et al., 2019). Does this bias toward the ensemble average influence grasping? Corbett and Song (2014) examined whether adaptation to two ensembles, presented on the left and right sides of the screen, which varied in average size would bias grasping behavior. The participants were cued to grasp one of two test dots which replaced the ensembles after an adaptation period. The non-grasped dot was the average size of all the dots in the adapting display, while the size of the target dot varied in set increments from the non-grasped dot. Participants completed their grasp by touching their fingers to the computer screen, matching the size of the 2D test dot, and then reported whether the test dot they “grasped” was larger or smaller than the non-grasped dot. The authors reported that perceptual judgments were biased as an inverse function of the average size of the adapting ensemble (i.e., a test dot adapted to a small average ensemble size was perceived as being larger than the non-grasped dot and vice versa). Additionally, the authors observed a perceptual bias in early but not late stages of grasping. Importantly, it is possible that grasping 2D targets permits relative visual processing to influence grip aperture. For example, grasps directed at 3D shapes appear to resist both Garner interference and Weber’s Law, but “grasps” directed at 2D shapes succumb to Garner interference and abide Weber’s Law (Holmes and Heath, 2013; Freud and Ganel, 2015; Ozana and Ganel, 2019). Conversely, in a visual crowding paradigm where participants were asked to make perceptual judgments and “grasps” toward a computer monitor where a 2D tilted bar surrounded by tilted flanker bars was presented, Bulakowski et al. (2009) found that perception integrated information from the surrounding flankers while the action did not. Because the 2D stimuli used in both studies likely did not fully engage the visuomotor system, it remains unknown whether ensemble perception can influence grasping under more ecologically valid circumstances. We have addressed this issue in the current study by using real 3D objects in our ensemble displays.

Importantly, in the studies discussed above, the grasping target consistently had an explicit size or orientation that the visuomotor system could utilize when planning and executing a grasping movement. Since grasping movements are tuned to the veridical dimensions of target objects, the visuomotor system efficiently controls movement by discounting irrelevant information when the properties of the target are explicitly available (Milner and Goodale, 2006). However, in cases where there is more ambiguity in a target object’s features, there may be more incentive for the visuomotor system to make use of all available perceptual information, including ensemble statistics. To this end, the present study aims to determine whether ensemble statistics bias grasping when visual information about the target is unconstrained and can afford a number of different grasp postures. Specifically, participants were tasked with grasping a circular 3D cylindrical target placed in the center of an ensemble display consisting of elliptical and circular cylinders. By using a single target of constant size and shape across the experimental session, we hoped to minimize scrutiny of the target and free up spatial attention to engage the ensemble statistics of the display. Although the size of the circular target was explicit and required a constrained grip aperture, the orientation of the grasp posture for a circular target was unconstrained, in that participants could place their fingers at almost any number of points along the circumference of the target to successfully grasp it. This allowed the orientation of the elliptical cylinders to generate perceptual biases that could conceivably bias the orientation of the grasp posture in favor of the ensemble mean orientation (note that this does not imply a conscious illusory percept of the target’s orientation, but rather a potential implicit bias of ensemble statistics on grasping behavior). We also varied the sizes of the ellipses in the ensemble, to determine whether the mean size of the ellipses could bias grip aperture. If ensemble processing can strongly influence grasping, then both grip orientation and grip aperture should be biased toward the mean orientation and size of the ensemble, respectively. If ensemble processing can only weakly affect grasping, then only grip orientation, in which the selection of grasp posture is relatively unconstrained, should be biased by ensemble processing. If grasping is not influenced by ensemble statistics, then we should observe no influence of mean orientation and size on grip orientation and grip aperture, respectively.

To isolate any obstacle avoidance effects on the grasping task, we administered a series of control trials in which the target was presented in isolation. If the non-target objects serve as obstacles to the central target, grip aperture would be expected to be smaller in the ensemble grasping task compared with the baseline grasping task (Bonfiglioli and Castiello, 1998; Chen et al., 2015). In a separate task, participants were asked to provide manual estimations of the perceived average size and orientation of the ensemble to ensure that the participants were able to perceive and report differences in ensemble statistical values. To make a manual estimate, participants separated their thumb and index finger to create a gap and oriented them in space so that these reports of size and orientation matched the average size and orientation of the ensemble. To ensure that the participants received the same haptic feedback for their perceptual estimates as they would for the grasping task, they were asked to grasp the target after completing their estimate. Based on the rich literature on the perceptual processing of ensemble statistics (see Whitney and Yamanashi Leib, 2018, for review), we hypothesized that within the perceptual-estimation session: (1) grip aperture (GA) should reflect perceived average ensemble size in the size-estimation task and (2) grip orientation (GO) should reflect perceived average ensemble orientation.

Taken together, the motivation for our study was to examine whether ensemble statistics, which are implicitly extracted and affect perception and memory (e.g., Brady and Alvarez, 2011; Sama et al., 2021), also affect visuomotor programming. Note that this differs from the design of visual-illusion paradigms that are used to investigate potential dissociations between action and perception, in that we are not concerned with whether ensemble statistics affect the perceived properties of the to-be-grasped target, but rather, whether such statistics implicitly bias grasping movements made toward that target. In a broader sense, we are interested in whether the statistics of the environment affect our everyday interactions with objects.

## Materials and Methods

### Participants

Fifteen participants (*M_age_* = 25.2, *SD_age_* = 4.54 years; 11 males), with normal or corrected-to-normal vision, were recruited from the University of Toronto community. Participants were right-handed as assessed by a modified version of the Edinburgh Handedness Inventory (Oldfield, 1971), and were compensated at a rate of $10 CAD/h for completing two, 1.5-h experimental sessions. Of note, 10 of these participants (*M_age_* = 25.2, *SD_age_* = 7.64 years; 4 males) took part in an initial version of the study, with the remaining five participants (*M_age_* = 24, *SD_age_* = 1 year; 4 males) taking part in a second round of data collection that entailed making slight modifications to the experimental procedure (see the “Modifications to the Experimental Procedure” section for more details). All participants gave informed consent prior to the start of the study. All experimental procedures were conducted in accordance with the University of Toronto Ethics Review Board.

### Stimuli

**Figure 1:**
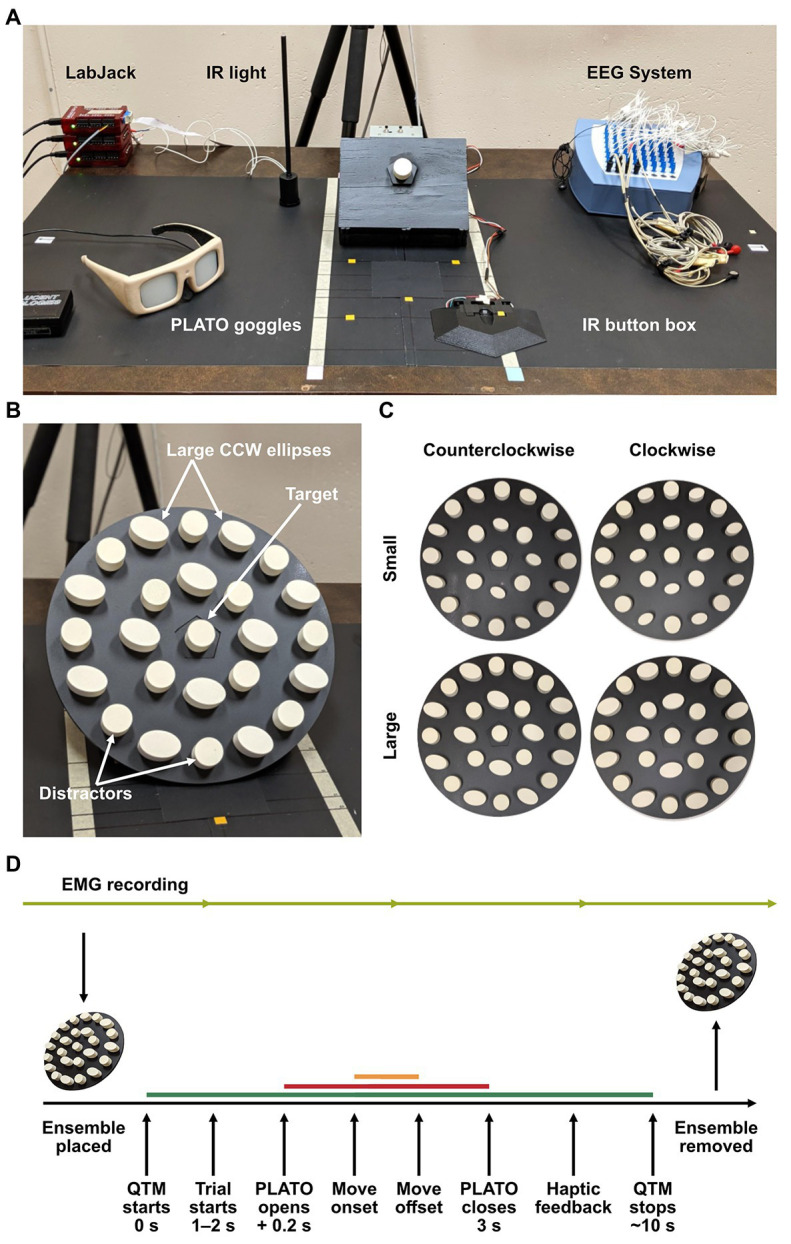
**(A)** Experimental set-up, **(B)** mounted ensemble display, and **(C)** ensemble display stimuli. The central target object was held in place on a peg in the center of a pentagonal base, which the displays were fitted on to. Cylindrical distractors labeled in **(B)** were interleaved between ellipses to prevent visual pop-out of the target. The four ensemble conditions of small × CW, large × CW, small × CCW, and large × CCW are photographed in panel **(C)**. This standardized the presentation of the displays and ensured that the target position was constant on all trials. **(D)** Experimental procedure for a single trial. After completing their manual estimation on perceptual trials, participants grasped the target object to ensure haptic feedback of the target was present in both grasping and perceptual-estimation sessions. Electromyography (EMG) was recorded continuously throughout the session (light green line). The motion capture recording length is depicted by the dark green line, the stimulus presentation is depicted by the red line, and movement duration by the orange line.

Each ensemble was made up of 25 objects that were designed with 3D-modeling software (*Blender*, version 2.79b, Windows. Blender Foundation, Amsterdam, the Netherlands) and printed in plastic using a 3D printer (ProJet® MJP 2500 Series, Objex Unlimited, Toronto, ON, Canada). The target object in the center of the display was shaped like a circular cylinder (diameter = 2.77 cm, height = 2.00 cm, and volume = 12.05 cm^3^). We used a single target size and location to encourage conditions of “kinematic consistency,” which have been shown to maximize the opportunity for ventral-stream involvement in grasping by minimizing the requirement for *de novo* dorsal-stream driven computations (Haffenden and Goodale, 2002). It was surrounded by 8 and 16 objects, evenly distributed 4.70 cm apart along two concentric rings 6 and 12 cm away from the middle, respectively (see Figure 1). Half the surrounding objects had the same size and shape as the target object so that it would not pop out visually. The other objects were elliptical cylinders. Their dimensions varied in three steps above and below a volume of 8.61 cm^3^ in the small ensemble size condition and 15.60 cm^3^ in the large ensemble size condition (see Table 1 for details). This resulted in a small average size ensemble display with an average volume of 10.33 cm^3^ and a large average size ensemble display with an average volume of 13.72 cm^3^. Orientations of the elliptical cylinders were also manipulated in three steps above and below 70° in the clockwise (CW) ensemble orientation condition and 110° in the counter-clockwise (CCW) ensemble orientation condition. This resulted in an average ensemble orientation of 70° in the CW condition and 110° in the CCW condition (circular cylinders did not convey orientation information; see Table 2). Each individual value of elliptical size and orientation (six each) was repeated twice within a given ensemble display (yielding 12 elliptical cylinders on each display), and specific size and orientation values were randomly assigned to positions on the inner (four ellipses) and outer (eight ellipses) rings of the ensemble displays. A square peg on the bottom of each cylinder fit into slots on an acrylic disk which ensured that the cylinders were accurately placed within the ensemble display. The entire display subtended approximately 24.5° of visual angle, and the inner ring subtended 9.9°.

**Table 1:** Size dimensions of cylinders in the ensemble displays.

Object size	Length (cm)	Width (cm)	Depth (cm)	Area (cm^2^)	Volume (cm^3^)
Circular cylinder	2.77	2.77	2.00	6.03	12.05
Small ellipse 1	1.77	2.66	2.00	3.70	7.40
Small ellipse 2	1.82	2.73	2.00	3.90	7.80
Small ellipse 3	1.87	2.80	2.00	4.10	8.21
Small ellipse 4	1.96	2.93	2.00	4.51	9.01
Small ellipse 5	2.00	3.00	2.00	4.71	9.42
Small ellipse 6	2.04	3.06	2.00	4.90	9.80
Small average size ensemble					10.33
Large ellipse 1	2.47	3.70	2.00	7.16	14.31
Large ellipse 2	2.50	3.74	2.00	7.33	14.67
Large ellipse 3	2.53	3.79	2.00	7.52	15.03
Large ellipse 4	2.59	3.88	2.00	7.87	15.75
Large ellipse 5	2.61	3.92	2.00	8.05	16.10
Large ellipse 6	2.64	3.97	2.00	8.23	16.46
Large average size ensemble					13.72

The dimensions of the elliptical cylinders varied in three steps above and below a volume of 8.61 cm^3^ in the small average size condition and 15.60 cm^3^ in the large average size condition. Each display held 12 elliptical cylinders, and as such each individual size was repeated twice within a display for its respective size condition.

**Table 2:** Orientation values of cylinders in the ensemble displays.

Object orientation	Degrees
Circular cylinder	NA
Clockwise ellipse 1	62.5
Clockwise ellipse 2	65
Clockwise ellipse 3	67.5
Clockwise ellipse 4	72.5
Clockwise ellipse 5	75
Clockwise ellipse 6	77.5
Clockwise average orientation ensemble	70
Counter-clockwise ellipse 1	102.5
Counter-clockwise ellipse 2	105
Counter-clockwise ellipse 3	107.5
Counter-clockwise ellipse 4	112.5
Counter-clockwise ellipse 5	115
Counter-clockwise ellipse 6	117.5
Counter-clockwise average orientation ensemble	110

Orientations of the elliptical cylinders were set to three steps above and below 70° in the clockwise average orientation condition and 110° in the counter-clockwise average orientation condition. Each display held 12 elliptical cylinders, and as such each individual orientation was repeated twice within a display for its respective orientation condition.

### Experimental Setup

Participants sat at a table with their right hand resting on an infrared (IR) button box positioned 18 cm from the table edge and 11.5 cm from the midline. Thirty centimeters directly in front of them was a display mount, tilted at a 45° angle, on which ensemble display disks could be interchanged. The target object was a cylinder held by a peg on a pentagonal platform in the center of the mount and was positioned 40 cm from the table edge. Behind the display and out of reach of the participant, a raised IR light acted as a timing flag for stimulus onset in the 3D-motion recording (see Figure 1A). Each ensemble disk had a pentagonal cut-out in the center, so it could be fitted on the mount in a specific and consistent orientation (see Figure 1B). There were five displays in total, one of which was an empty disk (i.e., no ensemble) that acted as a baseline measure for grasping. The other four varied in average size and orientation, resulting in (1) small/CW, (2) small/CCW, (3) large/CW, and (4) large/CCW ensemble display conditions (see Figure 1C). Liquid-crystal shutter goggles (PLATO system, Translucent Technologies Inc., ON, Canada) were worn to control stimulus presentation and were kept opaque while the ensemble displays were being set-up.

3D movement of the hand and fingers was recorded using three Qualisys Track Manager (QTM) camera units (Qualisys AB, Göteborg, Sweden). Surface electromyography (EMG) was recorded from five muscles (one shoulder muscle: anterior deltoid; and four forearm muscles: brachioradialis, common extensor digitorum, first dorsal interosseous, and the flexor digitorum profundus) using disposable surface electrodes (3 M Ag/AgCl, Red DotTM electrodes, 3 M Health Care, MN, United States) and a custom EEG set-up (ANT Neuro, Hengelo, The Netherlands) at a sampling frequency of 2,048 Hz. These target muscles are used in reach-to-grasp movements. Specifically, the anterior deltoid and brachioradialis support reaching and lifting movements, whereas the common extensor digitorum, first dorsal interosseous, and the flexor digitorum profundus contribute mainly to the precision grip (Kapandji, 1980; Maier and Hepp-Reymond, 1995; Bonnefoy et al., 2009). Additionally, the target muscles have been identified to be informative as an indirect measure of grip force (Hoozemans and Van Dieen, 2005; Lashgari et al., 2021). The EMG data were used only in the multivariate analysis. The experimental script was run on a Windows computer using MATLAB (version 2019b, MathWorks Inc., MA, United States). This computer was connected to a system of LabJack U3s (LabJack Corporation, CO, United States) that facilitated communication between the script, PLATO goggles, IR flag, IR button box, EEG system, and QTM cameras. Event flags were sent to the EEG system from the LabJack to synchronize EMG data with trial timing and 3D movement data. The timing lag of events and flags for all the equipment was measured to be <2.5 ± 3.28 ms on average, using an oscilloscope (PicoScope 2204A and software version 6, Pico Technology, England).

### Pilot Study

A pilot study was run to equate differences in perceptual sensitivity for object size and orientation features. Specifically, we aimed to ensure that the difference in perceptual sensitivity between two different physical values on the size continuum was equated to the difference in perceptual sensitivity between two different physical values on the orientation continuum. Participants were asked to rank the size or orientation of 16 elliptical cylinders, eight of which only varied in size (small to large), and eight that only varied in orientation (CW to CCW). The order of ranking size and orientation was counterbalanced across participants, and the presentation of the cylinders was pseudorandomized, such that each object was presented an equal number of times. The vertical axis was denoted as 90°, with any angle falling to the right being referred to as CW and angles greater than 90° or on the left side of the axis referred to as CCW. The smallest size and most CCW orientation were given a score of 1, and the largest size and most CW orientation a score of 8. Participants ranked objects one at a time and each object was ranked four times, with the resulting ranks being used to generate psychometric functions to guide the physical dimensions chosen for our ensemble stimuli. The ratio of the perceived rank to the actual rank of the objects was used to match the perceived difference between the large and small average ellipse size to the perceived difference between CW and CCW average orientation of our stimuli. This pilot procedure ensured that any difference between ensemble size and orientation processing in the main experiment could not be accounted for by differences in perceptual sensitivity across the stimulus features.

### 3D Motion Capture

The movement of the hand and fingers were recorded using eight passive IR markers attached to the inner corner of the nail for the thumb and index finger, the second joint of the index finger, first joint of the thumb, index, and little fingers, and the styloid process of the radius and ulna of the wrist. The main markers used in the univariate analysis were the markers on the distal phalanges of the thumb and index finger (the remaining six markers were used for multivariate analyses). Motion was recorded at a sampling rate of 240 Hz. Cameras were calibrated at the beginning of each session and at times when marker tracking became irregular (i.e., abnormal number of tracked markers displayed by the cameras).

### Procedure

The experiment was separated into grasping and perceptual-estimation sessions. In each session, the participant was instructed to perform different tasks; however, the general set-up and sequence of events were kept the same. All participants completed both sessions in 1 day, and session order was counterbalanced across participants. At the beginning of each trial, participants were required to rest their index finger on the IR button-box. This allowed us to precisely measure movement onset. The participant’s middle, ring, and little finger were secured using skin-friendly tape to ensure that they only grasped using their index finger and thumb. At the start of each trial, the ensemble disk corresponding to the condition on that trial (i.e., small/CW, small/CCW, large/CW, and large/CCW) would be mounted on the display, after which the experimenter would start the motion capture recording. After a random time interval between 1 and 2 s, the IR flag would turn on (this allowed us to align the trial data with a signal that was visible in the QTM data), and after 200 ms, the PLATO goggles would open. This marked the time of stimulus onset and acted as a go-signal for participants to begin their response. In the grasping session, the participant would pick up the target object (i.e., the central circular cylinder) using their thumb and index finger, place it back on the peg and return their index finger to the starting position. In the perceptual-estimation session, the participant would perform a manual-estimation task (i.e., judgments of either average size or orientation) by adjusting the distance between their thumb and index finger to report the perceived average size of the ensemble display and adjusting the angle between their thumb and index finger to report the perceived average orientation of the ensemble display. For both manual estimation tasks, participants were told to hold their fingers in place until the goggles closed before returning their hand to the starting position. In both sessions, the PLATO goggles remained open for 3 s (see Figure 1D). Participants were instructed to remain fixated on the central target object throughout the experiment, and although eye-tracking equipment was not used, fixation was monitored by the experimenter. If the participant made a faulty response, the incorrect display was used, or there were any major disturbances to the motion or EMG recordings (e.g., the cameras were disturbed, or the object dropped), the trial was excluded without replacement (which, including poor data recording, accounted for four trials on average per participant). At the beginning of each session, participants were given 10 practice trials to become accustomed to the task. In the grasping session, this consisted of grasping the central target surrounded by an ensemble display. In the perceptual session, the first half of the practice trials were matched to the estimation task they would encounter first in the experimental session, and the second half of the practice trials matched the other task. The presentation of ensemble displays was pseudorandomized such that each display was presented an equal number of times in the experimental trials and the same display was never shown twice in a row.

#### Perception – Manual Estimation Task

Participants gave manual perceptual estimations of average size and average orientation in separate blocks of trials. For the average size estimation task, participants were told to adjust the distance between the thumb and index finger of their right hand (keeping a constant orientation) until it matched their perceptual estimation of the average size of the ensemble objects. For the average orientation estimation task, they were told to rotate the imaginary line between their thumb and index finger (keeping the distance between the digits constant) until it matched their perceptual estimation of the average orientation of the ensemble objects. Participants were instructed to use the long axis of the ellipses for their responses in the size and orientation tasks.

Due to occlusion of the infrared-markers by the hand under certain movements, participants were asked to give their size estimation with their thumb and index finger orientated at a roughly constant 45°, and average orientation was always reported by turning the hand while holding the size of their GA constant. When reporting the CCW average ensemble orientation, participants were asked to rotate their hand CCW instead of CW as the latter resulted in the occlusion of the infrared markers. For both tasks, participants held their manual estimation until the goggles closed, after which they returned their hand to the starting position. In order to provide the same haptic feedback encountered in the grasping trials, the goggles then reopened, and the participant performed a typical grasp of the target before returning their hand to the starting position to await the start of the next trial. This haptic feedback was not recorded for the first set of participants, but the methods were modified afterward to include it (see “Modifications to the Experimental Procedure” section for more details). The session consisted of 90 trials, starting with 10 practice trials, followed by 80 experimental trials split into four blocks (two blocks each for the average size and orientation estimation tasks, the order of which was counterbalanced across participants using an ABAB design) of 20 trials each.

#### Action – Grasping Task

The opening of the goggles served as a “go-signal” for the participants, and they were instructed to reach for the central target object as quickly and accurately as possible, grasp it using a precision grip (between the thumb and index finger of the right hand), and lift it a short distance above the display before placing the object back and returning their hand to the start position. The session consisted of 110 trials, the first 10 being practice trials, followed by 10 baseline trials, 80 grasping trials (split into four blocks of 20 trials each, where each ensemble display was presented five times), and concluded with another 10 baseline trials. In the baseline trials, the central target object was presented alone on an empty display (i.e., with no surrounding ensemble). This allowed us to investigate the influence of obstacle avoidance on grasping targets embedded within an ensemble display and thus the baseline trials served as a control condition.

#### Modifications to the Experimental Procedure

After collecting data for 10 participants, we made two modifications to the experimental procedure based on methodological and theoretical considerations. First, we adjusted how the participants placed their fingers on the starting position at the beginning of each trial. For the first 10 participants, the hand starting position was a relaxed open palm with their right index finger in the IR button box. By using this starting position, the thumb and index finger were widely separated at the beginning of each trial, and as such maximum grip aperture (MGA) was flagged as occurring at the start of movement onset as the hand lifted from the table, instead of at roughly 75% of movement duration, as is typically reported with grasping tasks (Jeannerod, 1984; Hu and Goodale, 2000). Because of this, for the first 10 participants, we visually assessed the timepoint where MGA was found on a trial-by-trial basis, and where MGA occurred at movement onset, we manually adjusted the time interval to calculate MGA from 50 to 100% movement duration, to coincide with the standard procedure in the field. To avoid this unnecessary step for the final round of data collection, for the last five participants, we adjusted the starting position, so the thumb was touching the tip of the index finger as it rested on the button box. Second, we recorded and analyzed the haptic feedback component of the perceptual-estimation trials. That is, after participants made their manual estimation of either average size or orientation, they returned their thumb and index finger to the starting position, and then initiated a grasping movement to the central target, identical to the procedure used in the grasping trials. This allowed us to investigate whether making a prior perceptual estimation of either average size or orientation affected subsequent grasping movements to the target embedded within the ensemble display (in subsequent sections we refer to these distinctions as “perception,” “grasping,” and “haptic feedback”). We compared the data collected before and after these modifications and found that the data were very similar across both collection rounds except for GO during grasping (see “Data Collection Round – Original Experimental Procedure vs. Modified Experimental Procedure” section of the results for more details).

### Data Preprocessing

#### QTM Data Preprocessing

After data collection, the individual markers in the motion capture recordings were automatically labeled using a customized model in the Qualisys Track Manager software (version 1.8, Qualisys AB, Göteborg, Sweden). Each trial was then visually inspected and mislabeled markers were manually corrected. Missing coordinate data that spanned less than 20 frames (4.80 ms) were automatically gap-filled using non-uniform rational B-spline interpolation (Piegl and Tiller, 1987). Larger gaps were manually filled if they were near the start or end of the recording (before the go-signal or after the participant had returned their hand to the starting position). Otherwise, trials with large gaps were excluded. In a small number of trials, the recorded data were poor (e.g., 70+ recorded markers, instead of the expected nine). In these cases, it was not feasible to confidently individuate the markers and the trials were flagged for exclusion (including issues during data capture, this accounted for four trials on average per participant).

The pre-processed marker positions were then exported into MATLAB where velocity, peak velocity, onset and offset velocity, acceleration, stimulus onset time, movement onset and offset time, movement duration, reaction time, grip aperture, and grip orientation were defined. To reduce recording artifacts, the positional and velocity data were smoothed using low-pass Butterworth filters for position (*n* = 2, cut-off frequency = 8 Hz) and velocity (*n* = 2, cut-off frequency = 12 Hz), in the forward and backward direction to remove phase shift. The IR flag marker was used in conjunction with the timestamps collected in the trial data in the MATLAB experimental script to ensure that the timing of the MATLAB and QTM computer was aligned. Movement onset was defined as the time point when the index finger was lifted from the IR button box, movement offset was defined as when the velocity dropped below 10% of the peak velocity. Movement duration was defined as the time between movement onset and offset, and reaction time was defined as the time between stimulus onset and movement onset.

#### EMG Preprocessing

Asa (version 4.1, ANT Neuro, Hengelo, The Netherlands) was used to export the EMG files which were analyzed in MATLAB using Letswave6.^1^ The data were high pass filtered (Butterworth, *n* = 4, low cut-off frequency = 2 Hz) to remove artifacts and linear detrending and removal of DC offset were also applied to the data. Full-wave rectification of the signal was followed by smoothing with a low-pass Butterworth filter (*n* = 4, high cut-off frequency = 5 Hz) to construct the linear envelope. The signal was then down sampled to 240 Hz using the nearest neighbor interpolation method to match the sampling rate of the QTM data. The EMG signal was then aligned to movement onset and segmented from the session’s maximum reaction time to the maximum movement duration with a 200 ms buffer on either end.

### Data Exclusion and Cleaning

In addition to trials flagged for exclusion during data collection and preprocessing, trials were also excluded based on reaction time (<250 ms), percentage of total recording frames where the IR marker was tracked successfully (<95%), and the presence of outliers (beyond 1.5 times the interquartile range) for grip aperture or orientation. Based on these criteria, 16% of trials were excluded for the perception trials, 19% were excluded for the haptic feedback trials (recorded after modifications to the experimental procedure), and 11% were excluded for the grasping trials. This left a total of 1,007 trials for the perceptual data, 1,329 trials for the grasping data, and 324 trials for the haptic feedback data for the final analysis. Additionally, due to technical failure, the EMG data for one participant’s perception session could not be extracted.

## Data Analysis

### Univariate Analyses

#### Dependent Variables

Grip aperture was defined as the distance, in millimeters, between the thumb and index markers in 3D space during grasping or manual estimation. Grip orientation was defined as the angle between the horizontal axis of the ensemble display and the projection of the vector connecting the thumb and index fingers onto the display surface.

The dependent variables for the grasping and haptic feedback tasks were MGA and GO at the time of MGA, whereas the dependent variables for the perceptual manual estimation tasks were GA and GO at movement offset averaged over 16.7 ms (four frames). In the size-discrimination task, the relevant dependent measure was GA as this was scaled according to the participant’s estimate of average ensemble size. Likewise, GO was the relevant dependent measure in the orientation-discrimination task.

#### Perception – Manual Estimation Task

Multilevel models were used to model GA as a function of average ensemble size (small vs. large), average ensemble orientation (CW vs. CCW), the interaction between size and orientation, and the round of data collection (original experimental procedure vs. modified experimental procedure), for the size- and orientation-discrimination tasks separately. The intraclass correlation (ICC) for the models suggested that grip aperture was mildly clustered within participants (Size discrimination task: *ICC* = 0.38, *N* = 515, *α* = 0.05, *p* < 0.00001; Orientation discrimination task: *ICC* = 0.56, *N* = 492, *α* = 0.05, *p* < 0.00001), suggesting that it was appropriate to account for the interdependence of trial observations by including a random intercept for participant in our analysis. Grip orientation was modeled similarly, and the ICC for the models suggested that GO was significantly clustered within participants for the size-discrimination task (*ICC* = 0.720, *N* = 515, *α* = 0.05, *p* < 0.00001) but not for the orientation-discrimination task: *ICC* < 0.00001, *N* = 429, *α* = 0.05, *p* > 0.05). Therefore, the random intercept for participant was not strictly necessary in the orientation-discrimination task model.

To account for the nesting of trials within participants, all models included a random intercept for participant. Models were estimated with an unstructured covariance matrix using the lmer function from the lme4 package (version 1.1-23; Bates et al., 2015) in R version 3.6.2 (R Core Team, 2019). The lmerTest package (version 3.0-1; Kuznetsova et al., 2017) was used to report results of statistical tests including degrees of freedom which were estimated using Satterthwaite’s approximation. Effect sizes are reported as partial *R*^2^ values (Edwards et al., 2008).

#### Action – Grasping Task

For the grasping trials, MGA and GO were modeled separately as a function of average ensemble size, orientation, their interaction, and the round of data collection. Both grip aperture (*ICC* = 0.75, *N* = 1,081, *α* = 05, *p* < 0.00001) and grip orientation (*ICC* = 0.67, *N* = 1,081, *α* = 0.05, *p* < 0.00001) were moderately clustered within participants.

#### Grasping vs. Baseline vs. Haptic Feedback Tasks

To examine the influence of obstacle avoidance and prior perceptual processing on subsequent grasping movements, MGA and GO were modeled separately as a function of the type of grasping task (grasping vs. baseline vs. haptic feedback). The main grasping task was specified as the reference level, and so the baseline task was compared with the main grasping task to examine the effects of obstacle avoidance, while the haptic feedback task was compared to the main grasping task to examine effects of perceptual estimation on grasping. The ICC for both models suggested that both maximum grip aperture (*ICC* = 0.68, *N* = 1,653, *α* = 0.05, *p* < 0.00001) and grip orientation (*ICC* = 0.66, *N* = 1,653, *α* = 0.05, *p* < 0.00001) were moderately clustered within participants.

#### Variability of Grasping Movements

As grasps were directed to a single target that had a constant shape and size, it was possible that the grasping movement became stereotyped with repetition over the experimental session. In order to examine whether this occurred, we conducted a *post-hoc* analysis of the SD of MGA and GO across the grasping session (calculated for each participant; split into one bin of 10 practice trials, one bin of 10 initial baseline trials, four bins of 20 grasping trials each, and one bin of 10 final baseline trials). SD of MGA and GO were modeled separately as a function of trial bin, with the first bin (practice trials) as the reference level. The ICC for both models suggested that SD of MGA (*ICC* = 0.36, *N* = 1,446, *α* = 0.05, *p* < 0.00001) and GO (*ICC* = 0.56, *N* = 1,446, *α* = 0.05, *p* < 0.00001) were clustered within participants. All models included a random intercept for participant, and the dependent variable was modeled as a function of bin. Since the data for MGA and GO had slightly non-normally distributed residuals, we re-ran the analysis on square root transformed data. The results for GO did not differ from the original, and, while the transformed vs. non-transformed results were slightly different for MGA, the overall result was the same. That is, there was no decreasing trend of variability in grasping movements over time compared with the practice trials. Thus, we report the results using the non-transformed data for both GO and MGA, but the transformed results for both can be found in the Supplementary Materials.

### Multivariate Analyses

**Figure 2:**
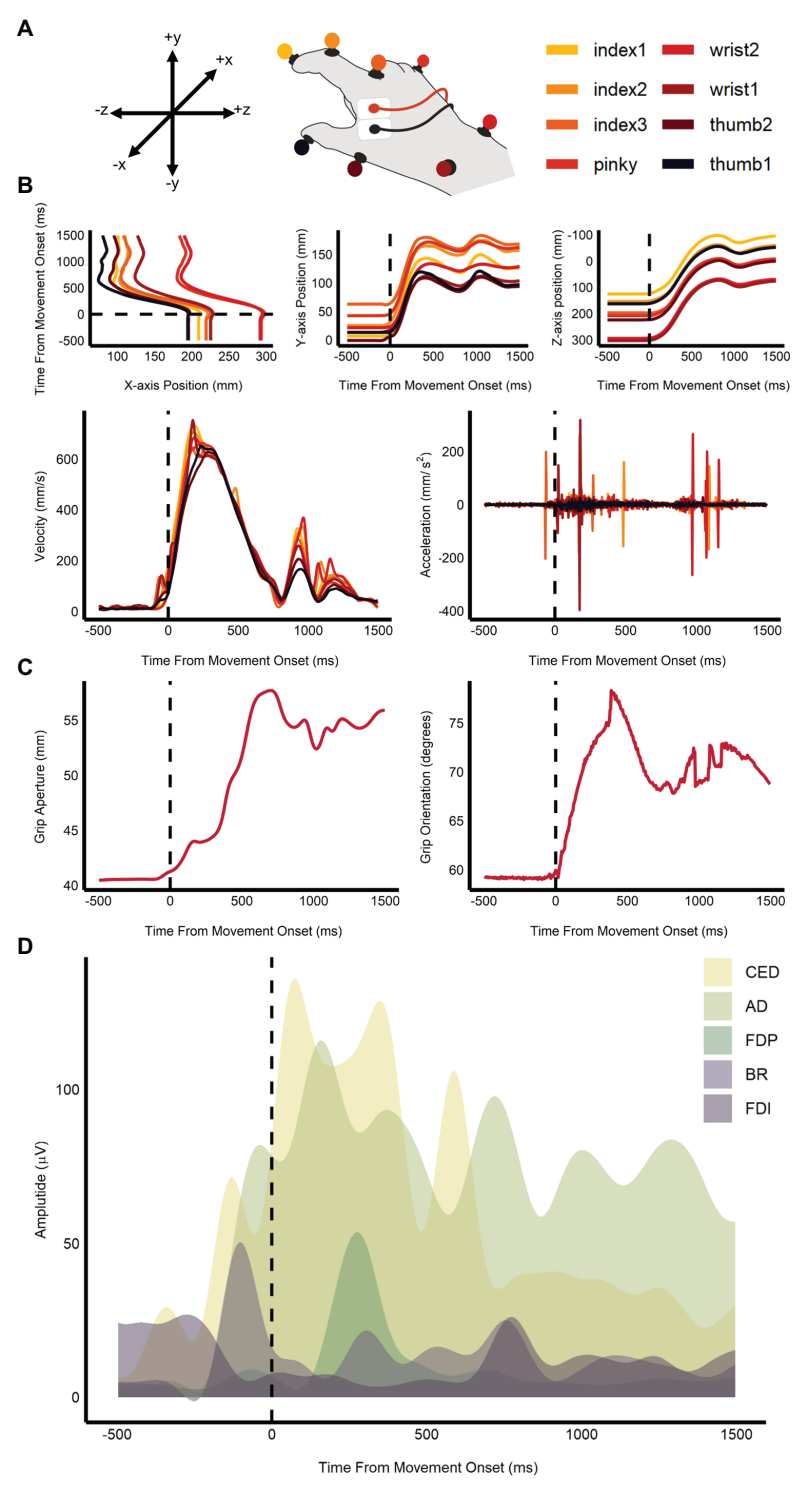
Sample of kinematic and EMG measures used in the multivariate analysis. Plot shows **(A)** the coordinate map of the *x*, *y*, and *z*-axes and an illustration of the markers’ position on the hand; **(B)** change in marker position, velocity, and acceleration over time; **(C)** change in grip aperture and orientation over time; and **(D)** plot of EMG amplitudes in the five recorded muscles: anterior deltoid (AD), brachioradialis (BR), common extensor digitorum (CED), first dorsal interosseous (FDI), and the flexor digitorum profundus (FDP) for one trial. The dashed line represents movement onset.

The QTM and EMG data were combined and used to train the support vector machine (SVM) models in the multivariate analysis. Specifically, this included the 3D coordinate data for all eight QTM markers, calculated velocity and acceleration, grip aperture, grip orientation, and the EMG channels from the five muscles (see Figure 2). SVM classification (LibSVM ver. 3.24, Chang and Lin, 2011) was used to determine whether average ensemble size (small vs. large) and orientation (CW vs. CCW) could be decoded from the kinematic and EMG data. SVM classification was performed across the timepoints between stimulus onset to movement offset (+200 ms on either end). Leave-one-out cross-validation was used to assess the performance of the classifier. Five classification permutations were performed for each participant. In each permutation, trials were randomized within their category and averaged in pairs. The averaged accuracy values for each participant were then calculated and combined. One-tailed one-sample *t*-tests, corrected for false discovery rate, were used to test if accuracy values at each time-point were significantly greater than chance.

## Results

### Univariate Results

#### Perception – Manual Estimation Task

**Figure 3:**
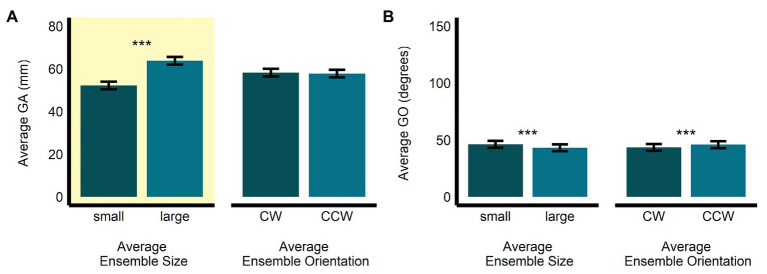
Univariate results of the perceptual average size-discrimination task. Estimated marginal means of average **(A)** grip aperture and **(B)** grip orientation are given for the small and large average size ensemble displays, and for the CW and CCW average orientation ensemble displays. ^***^p < 0.0001; error bars depict SEM; GA, grip aperture; GO, grip orientation; CW, clockwise; CCW, counter-clockwise. Grip aperture was the grasp parameter most relevant to the size-discrimination task and is highlighted in yellow.

In the size-discrimination task, there was a significant main effect of size (*b* = 11.60, *SE* = 0.53, *t*(497.52) = 21.76, *p* < 0.0001, *R*^2^ = 0.49) on GA. Grip aperture was the relevant dependent measure in the size-discrimination task, since changes in this measure were meant to scale with participants’ estimates of perceived average size. As expected, we observed that GA was significantly larger for the large average size ensemble displays (*M* = 63.8, *SE* = 1.79, 95% *CI* = [60.0, 67.7]) compared with the small average size displays (*M* = 52.2, *SE* = 1.8, 95% *CI* = [48.4, 56.1]). The main effect of orientation (*b* = −0.42, *SE* = 0.53, *t*(497.08) = −0.79, *p* = 0.431, *R*^2^ < 0.005; *M_CW_* = 58.2, *SE* = 1.79, 95% *CI* = [54.4, 62.1]; *M_CCW_* = 57.8, *SE* = 1.80, 95% *CI* = [54.0, 61.7]; see Figure 3A) and the interaction between size and orientation were not significant (*b* = 0.53, *SE* = 1.07, *t*(496.95) = 0.50, *p* = 0.619, *R*^2^ < 0.00001). There was a significant main effect of size (*b* = −3.02, *SE* = 0.58, *t*(496.94) = −5.24, *p* < 0.0001, *R*^2^ = 0.05), and orientation (*b* = 2.39, *SE* = 0.58, *t*(496.74) = 4.15, *p* < 0.001, *R^2^* = 0.03), on GO in the size-discrimination task. The results indicated that GO was larger in the small average size display (*M* = 46.3, *SE* = 3.04, 95% *CI* = [39.7, 52.8]) than in the large average size display (*M* = 43.2, *SE* = 3.03, 95% *CI* = [36.7, 49.8], and it was also larger in the CCW average orientation display (*M* = 45.9, *SE* = 3.04, 95% *CI* = [39.4, 52.5]) compared with the CW orientation display (*M* = 43.6, *SE* = 3.03, 95% *CI* = [37.0, 50.1]; see Figure 3B). The interaction between size and orientation was not significant (*b* = 0.59, *SE* = 1.15, *t*(496.68) = 0.51, *p* = 0.609, *R*^2^ < 0.005).

**Figure 4:**
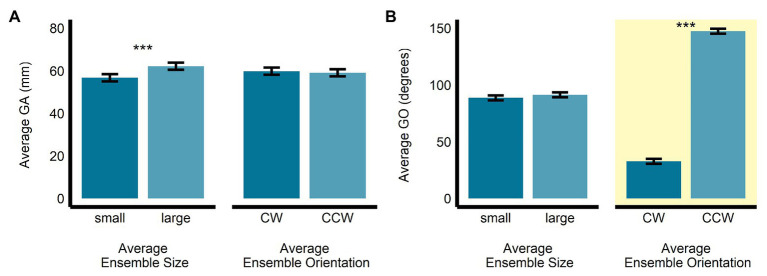
Univariate results of the perceptual average orientation-discrimination task. Estimated marginal means of average **(A)** grip aperture and **(B)** grip orientation are given for the small and large average size ensemble displays, and for the CW and CCW average orientation ensemble displays. ^***^p < 0.0001; error bars depict SEM; GA, grip aperture; GO, grip orientation; CW, clockwise; CCW, counter-clockwise. Grip orientation was the grasp parameter most relevant to the orientation-discrimination task and is highlighted in yellow.

In the orientation-discrimination task, there was a significant main effect of size (*b* = 5.43, *SE* = 0.42, *t*(474.22) = 12.86, *p* < 0.0001, *R*^2^ = 0.26) on GA. Specifically, GA was significantly larger in the large average size display (*M* = 62.2, *SE* = 1.69, 95% *CI* = [58.6, 65.8]) compared with the small average size display (*M* = 56.8, *SE* = 1.69, 95% *CI* = [53.1, 60.4]). The main effect of orientation (*b* = −0.73, *SE* = 0.42, *t*(474.24) = −1.73, *p* = 0.085, *R*^2^ = 0.01; *M_CW_* = 59.9, *SE* = 1.69, 95% *CI* = [56.2, 63.5]; *M_CCW_* = 59.1, *SE* = 1.69, 95% *CI* = [55.5, 62.8]; see Figure 4A) and the interaction between size and orientation were not significant (*b* = 0.01, *SE* = 0.84, *t*(474.02) = 0.08, *p* = 0.941, *R*^2^ < 0.00001). There was a significant main effect of orientation (*b* = 114.58, *SE* = 2.03, *t*(478.38) = 56.35, *p* < 0.0001, *R*^2^ = 0.87), but not size (*b* = 2.72, *SE* = 2.03, *t*(477.82) = 1.34, *p* = 0.181, *R*^2^ < 0.005) on GO in the orientation-discrimination task. The interaction between size and orientation was significant (*b* = 13.48, *SE* = 4.05, *t*(475.51) = 3.33, *p* < 0.001, *R*^2^ = 0.02). Grip orientation was the relevant dependent measure in the orientation-discrimination task, since changes in this measure were meant to scale with participants’ estimates of perceived average orientation. As expected, GO was significantly larger in the CCW average orientation display (*M* = 147.4, *SE* = 2.22, 95% *CI* = [142.7, 152.0]) than in the CW average orientation display (*M* = 32.78, *SE* = 2.18, 95% *CI* = [28.23, 37.33]). GO did not significantly differ between the small (*M* = 88.7, *SE* = 2.2, 95% *CI* = [84.1, 93.3]) and large (*M* = 91.4, *SE* = 2.2, 95% *CI* = [86.8, 96.0]) average size displays (see Figure 4B).

**Figure 5:**
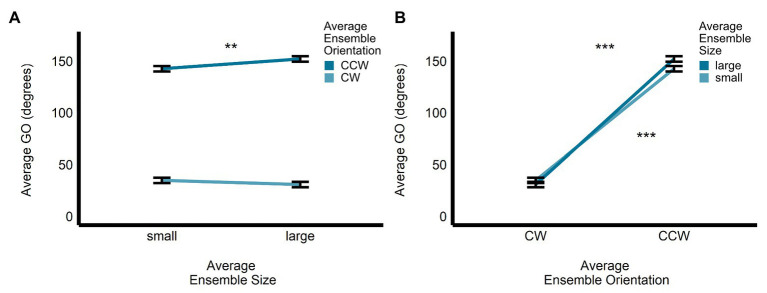
Univariate results of the size-by-orientation interaction for GO in the perceptual orientation-discrimination task. Differences in the estimated marginal means of GO are plotted for **(A)** small vs. large average size, for CW and CCW ensemble displays, and **(B)** CW vs. CCW average orientation, for small and large ensemble displays. ^**^p < 0.005, ^***^p < 0.0001; error bars depict SEM; GO, grip orientation; CW, clockwise; CCW, counter-clockwise.

Further examination of the significant interaction between size and orientation revealed that GO was significantly larger for the large compared with the small ensembles for the CCW average orientation displays (*b* = −9.46, *SE* = 2.94, *t*(476) = −3.22, *p* < 0.005), but not for the CW average orientation displays (*b* = 4.02, *SE* = 2.79, *t*(478) = 1.44, *p* = 0.15, *R*^2^ < 0.005; see Figure 5A). In contrast, the significant difference in GO between CW and CCW ensemble displays was apparent for both the small (*b* = −1.08, *SE* = 0.88, *t*(477) = −37.43, *p* < 0.0001) and large (*b* = −121, *SE* = 2.86, *t*(477) = −42.46, *p* < 0.0001) average size ensemble displays (see Figure 5B).

#### Action – Grasping Task

**Figure 6:**
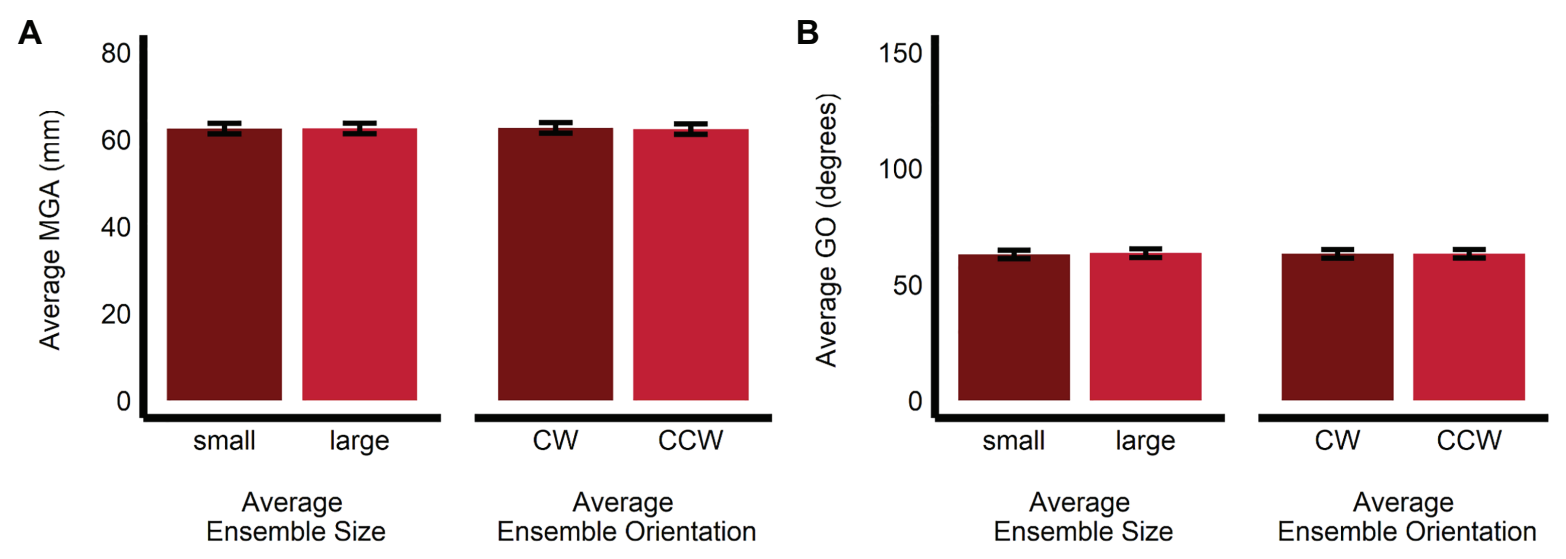
Univariate results of the grasping task. Estimated marginal means of average **(A)** maximum grip aperture and **(B)** grip orientation are given for the small and large average size ensemble displays, and for the CW and CCW average orientation ensemble displays. Error bars depict SEM; MGA, maximum grip aperture; GO, grip orientation; CW, clockwise; CCW, counter-clockwise.

Maximum grip aperture was not significantly affected by average ensemble size (*b* = 0.02, *SE* = 0.16, *t*(1063.01) = 0.148, *p* = 0.88, *R*^2^ < 0.001; *M_Large_* = 62.5, *SE* = 1.2, 95% *CI* = [59.9, 65.1]; *M_Small_* = 62.5, *SE* = 1.2, 95% *CI* = [59.9, 65.1]), orientation (*b* = −0.29, *SE* = 0.16, *t*(1063.02) = −1.86, *p* = 0.064, *R*^2^ < 0.005; *M_CCW_* = 62.4, *SE* = 1.2, 95% *CI* = [59.8, 65.0]; *M_CW_* = 62.7, *SE* = 1.2, 95% *CI* = [60.1, 65.3]), or their interaction (*b* = −0.03, *SE* = 0.31, *t*(1,063) = −0.09, *p* = 0.93, *R*^2^ < 0.001; see Figure 6A).

Similarly, GO was not significantly affected by average ensemble size (*b* = 0.50, *SE* = 0.38, *t*(1,063) = 1.34, *p* = 0.18, *R*^2^ < 0.005; *M_Large_* = 63.5, *SE* = 1.36, 95% *CI* = [59.5, 67.5]; *M_Small_* = 63.0, *SE* = 1.86, 95% *CI* = [59.0, 67.0]), orientation (*b* = 0.07, *SE* = 0.38, *t*(1,063) = 0.20, *p* = 0.84, *R*^2^ < 0.001; *M_CCW_* = 63.3, *SE* = 1.86, 95% *CI* = [59.3, 67.3]; *M_CW_* = 63.2, *SE* = 1.86, 95% *CI* = [59.2, 67.2]; *b* = 0.07, *SE* = 0.38, *t*(1,063) = 0.20, *p* = 0.84, *R*^2^ < 0.001), or their interaction (*b* = 0.51, *SE* = 0.75, *t*(1,063) = 0.68, *p* = 0.50, *R*^2^ < 0.001; see Figure 6B).

#### Data Collection Round – Original Experimental Procedure vs. Modified Experimental Procedure

The effect of round of data collection (original procedure vs. modified procedure; see “Modifications to the Experimental Procedure” for more details) was not significant for both GA and GO in the perceptual manual-estimation task (size-discrimination task: *b_GA_* = −0.91, *SE* = 3.55, *t*(12.98) = −0.26, *p* = 0.81, *R*^2^ < 0.01, *b_GO_* = 2.4, *SE* = 6.05, *t*(12.71) = 0.40, *p* = 0.698, *R*^2^ = 0.01; orientation-discrimination task: *b_GA_* = −1.13, *SE* = 3.55, *t*(12.92) = −0.34, *p* = 0.74, *R*^2^ = 0.01, *b_GO_* = −1.26, *SE* = 3.91, *t*(12.76) = −0.31, *p* = 0.76, *R*^2^ = 0.01).

For the grasping trials, the effect of round of data collection was not significant for MGA (*b* = −2.66, *SE* = 2.40, *t*(13.0) = −1.11, *p* = 0.29, *R*^2^ = 0.09), but there was a significant effect on GO (*b* = 12.42, *SE* = 3.70, *t*(12.99) = 3.36, *p* < 0.05, *R*^2^ = 0.46). Together, this demonstrates that, by and large, the modifications we made to our experimental procedure did not appreciably affect the main results of our univariate analyses.

#### The Effect of Obstacle Avoidance and Perceptual Processing on Grasping Movements

As we stated above, to examine the influence of obstacle avoidance and prior perceptual processing on subsequent grasping movements, we compared the data in the main grasping trials with those in the baseline grasping (i.e., grasping the central target in the absence of a surrounding ensemble) and haptic feedback (i.e., conducting a grasping movement immediately after making a discrimination of average size or orientation in the perceptual manual-estimation task) trials, respectively. We found that there was a significant main effect of task on MGA (*F*(2, 1638.12) = 164.84, *p* < 0.0001, *R*^2^ = 0.17) and GO (*F*(2, 1688.70) = 6.95, *p* < 0.001, *R*^2^ = 0.01).

**Figure 7:**
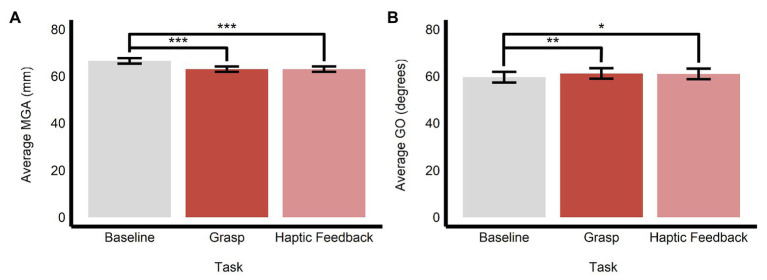
Effects of obstacle avoidance and prior perceptual processing on grasping movements. Estimated marginal means of **(A)** maximum grip aperture and **(B)** grip orientation are plotted for the three different kinds of grasping tasks employed in this study. Comparing the baseline and grasp tasks reveals the effects of obstacle avoidance, whereas comparing the grasp and haptic feedback tasks reveals the effects of prior perceptual processing on subsequent grasping movements. ^*^p < 0.05, ^**^p < 0.005, ^***^p < 0.0001; error bars depict SEM; MGA, maximum grip aperture; GO, grip orientation.

Specifically, the baseline task (*M* = 66.5, *SE* = 1.16, 95% *CI* = [64.0, 69.0]) had significantly larger values of MGA compared with both the main grasping task (*M* = 63.0, *SE* = 1.15, 95% *CI* = [60.5, 65.4]; *b* = 3.52, *SE* = 0.20, *t*(1,636) = 17.85, *p* < 0.0001) and haptic feedback task (*M* = 63.0, *SE* = 1.16, 95% *CI* = [60.5, 65.5]; *b* = 3.49, *SE* = 0.26, *t*(1,638) = 13.55, *p* < 0.0001). MGA in the grasping and haptic feedback tasks did not significantly differ (*b* = −0.03, *SE* = 0.21, *t*(1,640) = −0.15, *p* = 0.987; see Figure 7A). Similarly, grip orientation was significantly smaller (more CW) in the baseline task (*M* = 59.6, *SE* = 2.26, 95% *CI* = [54.7, 64.4]) compared with both the grasping (*M* = 61.2, *SE* = 2.23, 95% *CI* = [56.4, 66.0]; *b* = −1.62, *SE* = 0.44, *t*(1,636) = −3.72, *p* < 0.001) and haptic feedback (*M* = 61.0, *SE* = 2.26, 95% *CI* = [56.1, 65.8]; *b* = −1.41, *SE* = 0.5, *t*(1,639) = −2.48, *p* < 0.05) tasks. Like the results with MGA above, GO did not significantly differ between the grasping and haptic feedback tasks (*b* = 0.21, *SE* = 0.46, *t*(1,641) = 0.46, *p* = 0.891; see Figure 7B).

#### Variability of Grasping Movements

**Figure 8:**
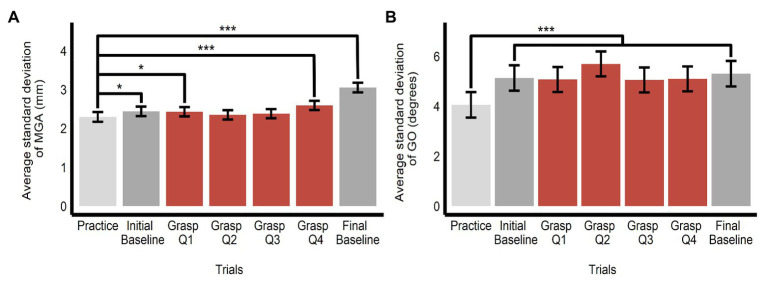
Variability of grasping movements. Estimated marginal means of average SD of **(A)** maximum grip aperture and **(B)** grip orientation across the grasping trials (split into one bin of 10 practice trials, one bin of 10 initial baseline trials, four bins of 20 grasping trials each (Q1–Q4), and one bin of 10 final baseline trials). Comparisons are made between practice trials and all other bins. ^*^p < 0.05, ^***^p < 0.0001; error bars depict SEM; MGA, maximum grip aperture; GO, grip orientation.

Because grasping movements might have become stereotyped with repetition, thereby obscuring any influence of the ensembles, we tested whether variability declined over time, by comparing variability in each grasping bin to that observed in the practice bin. However, we observed no such trend (in fact, the data trended in the opposite direction, with some bins showing significantly greater variability compared with the practice trials). There was an overall effect of bin on the SD of MGA (*F*(6, 1425.32) = 29.89, *p* < 0.0001, *R*^2^ = 0.11) and GO (*F*(6, 1425.15) = 13.82, *p* < 0.0001, *R*^2^ = 0.06). The SD of MGA was smaller in the practice trials (*M* = 2.30, *SE* = 0.12, 95% *CI* = [2.04, 2.56]) compared to the baseline trials (*M_Initial baseline_* = 2.44, *SE* = 0.12, 95% *CI* = [2.18, 2.70]; *b* = 0.14, *SE* = 0.07, *t*(1,425) = 1.99, *p* = 0.047; *M_Final baseline_* = 3.05, *SE* = 0.12, 95% *CI* = [2.79, 3.31]; *b* = 0.76, *SE* = 0.07, *t*(1,425) = 10.49, *p* < 0.0001) and the 1st and 4th quartile of the grasping trials (*M_Grasp bin1_* = 2.43, *SE* = 0.12, 95% *CI* = [2.18, 2.68]; *b* = 0.13, *SE* = 0.06, *t*(1,426) = 2.15, *p* = 0.032; *M_Grasp bin 4_* = 2.59, *SE* = 0.12, 95% *CI* = [2.34, 2.84]; *b* = 0.30, *SE* = 0.06, *t*(1,425) = 4.77, *p* < 0.0001; see Figure 8A). The SD of GO was smaller in the practice trials (*M* = 4.06, *SE* = 0.51, 95% *CI* = [2.97, 5.14]) compared with all other baseline and grasping bins (all *t*s > 5.05, all *p*s < 0.0001; see Figure 8B).

### Multivariate Results

**Figure 9:**
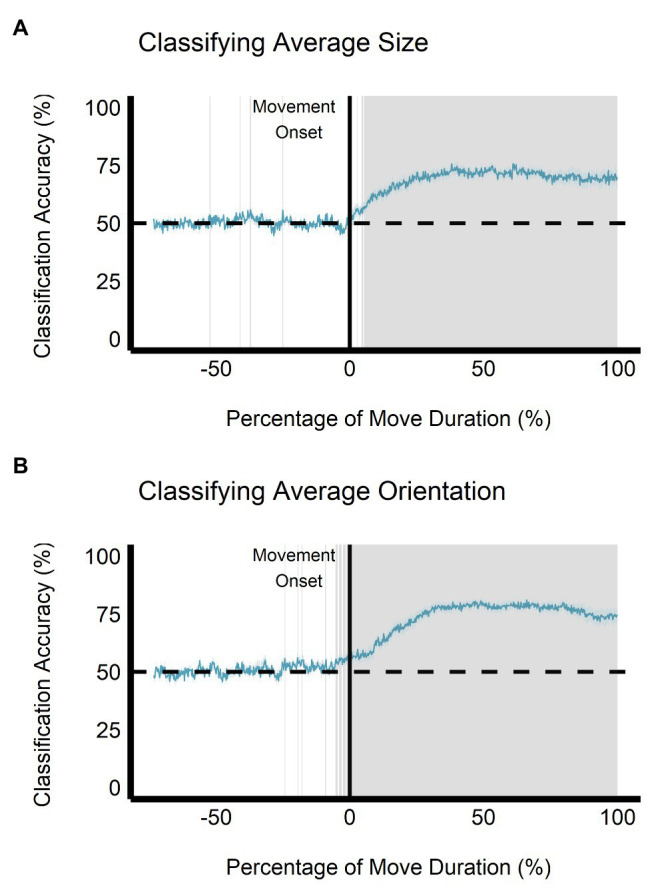
Multivariate results of the perceptual manual-estimation task. Support vector machine (SVM) classification accuracy is plotted for discriminations of **(A)** average ensemble size (small vs. large) and **(B)** average ensemble orientation (CW vs. CCW) across the duration of movement. Negative percentage values of movement duration show baseline accuracy values beginning at stimulus onset. The gray shaded area represents timepoints where SVM classification accuracy is significantly different than chance level after correcting for false discovery rate. Gray shaded rectangles = *p* < 0.05; Light blue region = depicts SEM.

**Figure 10:**
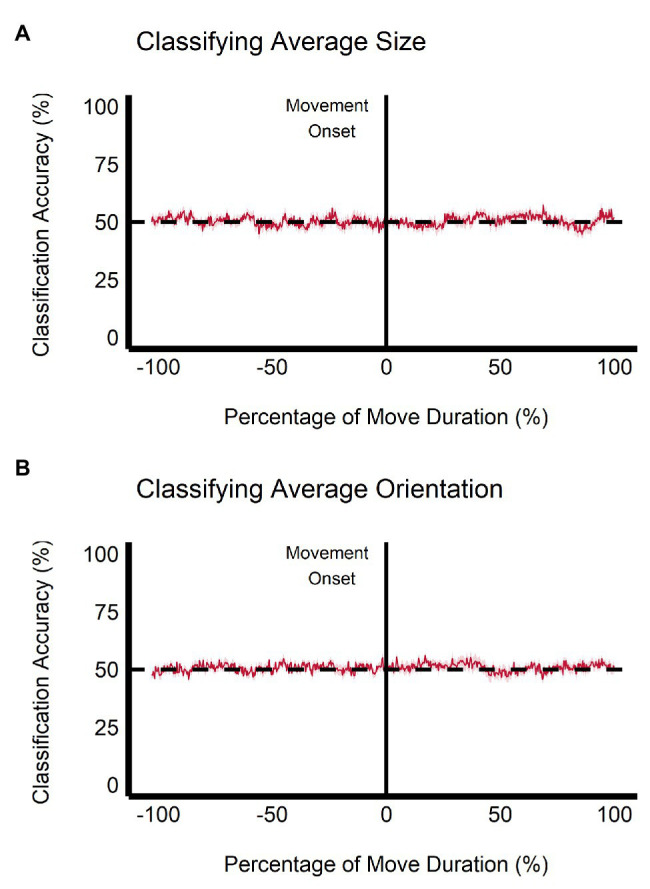
Multivariate results of the grasping task. SVM classification accuracy is plotted for discriminations of **(A)** average ensemble size (small vs. large) and **(B)** average ensemble orientation (CW vs. CCW) across the duration of movement. Negative percentage values of movement duration show baseline accuracy values beginning at stimulus onset. Light red region = depicts SEM.

When trained on the perceptual data, the classification accuracy between the small and large ensemble size begins to increase after movement onset, becoming significantly greater than chance level (50%) after 112.50 ms (approximately 5.34% of movement duration) and remains significantly greater than chance throughout the rest of the movement duration (see Figure 9A). This change is not seen when the classifier is trained and tested on the grasping data as the classification accuracy remains at chance level throughout movement duration (see Figure 10A). Classification accuracy of the haptic feedback trials did not differ significantly from chance, except for one timepoint (accounting for 4.16 ms) occurring at 40.04% movement duration.

Similarly, when trained on the perceptual data, classification of ensemble orientation (i.e., CW vs. CCW) becomes significantly greater than chance level 25 ms before movement onset (−1.19% of movement duration) and remains significantly greater than chance throughout movement duration (see Figure 9B). In contrast, when the classifier is trained on the grasping data, classification accuracy does not significantly differ from chance throughout the movement duration (see Figure 10B). Classification accuracy of the haptic feedback trials did not significantly differ from chance at any timepoint.

## Discussion

In this study, we investigated whether ensemble statistics can bias grasping behavior. We did this by asking participants to reach out to grasp a target circular cylinder that was surrounded by a background ensemble of circular and elliptical cylinders that varied systematically in their orientation and size. Notably, unlike ellipses, circular targets afford multiple uniquely comfortable or efficient grasp postures, meaning that participants would be free to orient their grip however they saw fit (in other words, the circular target did not constrain a specific grasp posture with respect to grip orientation). We reasoned that this uncertainty may render the visuomotor system more susceptible to the influence of ensemble perceptual processing. Furthermore, it is also conceivable that the visuomotor system might be biased by the mean size of the ensemble, even though a smooth and successful grasp requires that grip aperture remain tuned to the real size of the target.

Using traditional univariate techniques and more powerful SVM multivariate statistical models, we found that both grip orientation and maximum grip aperture were not influenced by ensemble perception. This was true even on trials where participants provided a manual estimate of average size or orientation before grasping the target (haptic feedback trials). Importantly, the visuomotor system’s insensitivity to ensemble statistics cannot be due to a failure of our setup to induce standard ensemble perceptual effects, because, in a separate block of trials, both ensemble-mean size and orientation significantly biased perceptual estimates of the average size and orientation of the ensemble. Additionally, haptic feedback from the target cannot be responsible for rectifying the perceptual effects, in a general sense, because we observed perceptual sensitivity to average size and orientation despite providing the participants an opportunity to grasp the target after each estimate.

### Perceptual Estimations

The univariate results revealed that, as predicted, perceptual estimates of average size and orientation were biased toward the average ensemble size and orientation, respectively, of the ensemble displays. Specifically, in the size-matching task, GA was wider when the average ensemble size was large (and smaller when the average ensemble size was small). In the orientation-matching task, GO was more CW when the average ensemble orientation was CW (and more CCW when the average ensemble orientation was CCW).

In testament to the strength of the bias induced by ensemble perception, we observed effects of the *non-relevant* ensemble property on the estimates. Thus, in addition to the bias of ensemble size on the size estimates, participants demonstrated bias in the *orientation of their estimates* toward the mean orientation of the ensemble when reporting average size. Furthermore, in addition to the bias of ensemble orientation on orientation estimates, participants demonstrated bias in the *size of their estimates* toward the mean size of the ensemble when reporting average orientation. Remarkably, these effects occurred despite our instruction to the participants to focus only on the task-relevant property of the target (size or orientation) and reinforce the view that ensemble perception is a holistic, rather than analytical, process that emerges out of entrenched structures that are largely refractory to knowledge, much like the phenomenology of pictorial illusions (see “Grasping” section below for a discussion of how our paradigm differs from those using visual illusions to investigate perceptual effects on grasping). Indeed, parallel summary representations for visual features outside the focus of attention have been demonstrated previously (Alvarez and Oliva, 2008, 2009; Emmanouil and Treisman, 2008; Attarha and Moore, 2015; Yörük and Boduroglu, 2020), and the implicit processing of one summary feature can bias the explicit processing of another (Sama et al., 2021).

We also observed that GO was more CCW in the small ensemble size than the large ensemble size in the size-discrimination task. This was unexpected as the bias toward the CW and CCW ensemble orientations would be averaged across size (i.e., the small average size ensembles included both the small × CW and small × CCW displays) and should result in similar values for GO across ensemble size. In fact, that is what we observed in the orientation-estimation task where no significant difference in GO between the small and large average ensemble size was observed. Upon further examination of the pairwise comparisons, we found that GO was larger for the large size than the small average ensemble size for the CCW but not the CW displays. This result was unexpected given that GO should be largely independent of ensemble size. More research might be required to better understand the effect observed.

Additionally, we observed that classification accuracy achieved above chance levels when classifying perceptual estimations of average size and orientation. These results suggest that ensemble size and orientation can be decoded from the kinematic and EMG data of the perceptual trials, and together with the results of the univariate analysis, demonstrate that participants were quite sensitive to perceptual differences of ensemble statistical values in our displays.

### Grasping

In contrast to the perceptual estimates, the univariate analysis of grasping did not detect any bias of ensemble size and orientation on maximum grip aperture and grip orientation, respectively. The failure of ensemble perception to influence grip aperture is consistent with studies which report that this measure resists the perceptual bias induced by pictorial illusions on targets embedded in them (Brenner and Smeets, 1996; Haffenden and Goodale, 1998; Jackson and Shaw, 2000; Servos et al., 2000; Danckert et al., 2002; Chen et al., 2015; Knol et al., 2017). Note, however, an important distinction between our paradigm and those used when studying the effects of visual illusions on grasping. In the latter, it is important to demonstrate that the illusion affects the perceived properties of the to-be-grasped target, to put any effect (or lack thereof) of the illusion on grasping into context. In our paradigm, we were not concerned with whether ensemble statistics have a direct effect on the perception of the circular target, which might then bias the grasps, but rather, whether such statistics, which are implicitly extracted and inform perceptual judgment, estimation, and memory (e.g., Brady and Alvarez, 2011; Sama et al., 2021), can inform the parameterization of the details of reach-to-grasp actions, particularly when the grasp conditions, a same-sized target disk with multiple grip posture affordances, ostensibly favor a computationally efficient resolution using ensemble summary statistics. In this sense, our purpose is not to investigate dissociations between perception and action *per se*, but to test whether implicitly extracted perceptual information of the surrounding scene biases how we interact with objects within that scene.

Although our univariate analyses allowed for a direct comparison of the most relevant grasp variables, it is limited by data averaging and by its nature is unable to reveal complex relationships between multiple variables. To circumvent this issue, and to provide complementary evidence for our univariate results, we examined grasping behavior (and perceptual estimations) using a more powerful multivariate analysis, which included both the kinematic and EMG data. This analysis still failed to detect any influence of mean ensemble size and orientation on multiple visuomotor measures (i.e., MGA, GO, velocity, acceleration, and the EMG data). These findings are in line with that of Bulakowski et al. (2009) who found that information from the surround was incorporated in the perception of, but not actions directed toward, an oriented target bar in a visual crowding paradigm. The multivariate techniques were applied across the time course of the reach, and we did not detect any change in classification accuracy. Thus, our time-course analysis does not support a distinction between early and late stage visuomotor processing as predicted with the planning and control model of visuomotor function (Glover and Dixon, 2002; Glover, 2004). Taken together, these results strongly indicate that ensemble perception does not bias grasping movements.

### Features and Limitations of the Design

An important aspect of ensemble perception, ensemble summary statistics, and the representations that underlie them, is that they are generated independently of the requirement to respond. Thus, the simultaneous presentation format was an important feature of our design, in which ensemble representations could be generated using all items of the ensemble before the participant responds. This feature of our design differentiates our study from a recent study by Hamidi et al. (2021), who similarly sought to test ensemble summary statistics on perception and action. In Hamidi et al.’s design, on each trial, participants were presented with only a single target object and, depending on the condition, either estimated its size or reached to grasp it. Given this serial presentation format and the inter-trial-intervals that are necessarily involved, this meant that the time required to generate an ensemble representation was on the scale of minutes, rather than the more typical scale of seconds or fractions of a second used here and elsewhere. Notably, previous experiments designed to manipulate ensemble statistical summary generation in the temporal domain have relied on rapid serial visual presentation, in which participants view all of the items of the ensemble within a few seconds (Chong and Treisman, 2003; Leib et al., 2014; Ying et al., 2020). This difference in time scales suggests the recruitment of different memory systems. Moreover, it is not clear how the requirement to respond to each one of the items interacts with the processes under investigation. Specifically, it is not clear whether Hamidi et al. (2021) manipulated an ensemble representation, as we conceive it, or if they manipulated learned stimulus-response mappings. The latter can be conceptualized, for example, as a shifted prior probability governing the relationship between visible target size and response output. One way to disentangle these two ideas would have been to test for after-effects. Updates to underlying stimulus-response priors, for example, should persist for several trials after the prior-shifting influence has been withdrawn, whereas the influence of an ensemble statistical summary should behave more transiently. In short, it is not clear whether Hamidi et al.’s operational use of the term “ensemble” is comparable to ours.

Another feature of our design is the use of real 3D objects and “real-time” visual conditions, because we were interested in testing ensemble statistical influence on dorsal-stream driven grasps. As we pointed out in the introduction, real-time 3D visual and haptic feedback are foundational conditions for typical dorsal-stream driven goal-directed action. Furthermore, our choice of using a single sized-target was designed to encourage ventral-stream engagement, thus maximizing our chances of observing an effect of perceptual ensemble representations on grasping behavior. Given all of these considerations, our results uniquely demonstrate the insensitivity of grasping movements to ensemble statistics using a more powerful multivariate analytical technique, incorporating multidimensional kinematic and EMG data.

A possible limitation of our study is that only one target object was used. Although this was an important feature of our design, geared to promote ventral-stream influence on grasping, it could also have contributed to grasping movements becoming highly stereotyped with repetition. To test for this, we conducted a *post-hoc* analysis to investigate whether the SD of MGA or GO changed significantly throughout the grasping session. If grasping behavior was becoming more practiced, there should be a reduction in variability over time. However, our results did not show a monotonic decline across the grasping session, demonstrating that grasping behavior did not become increasingly stereotyped from the initial practice trials onward. The visual illusion and grasping literature have also examined whether illusory effects on grasping persist with repeated grasping movements. On the one hand, some suggest that the effects of visual illusions on grasping gradually decrease as grasps are repeated (Cesanek et al., 2016; Whitwell et al., 2016). On the other hand, some suggest that the illusory effects remain constant throughout the experiment (Franz et al., 2001; Kopiske et al., 2016). Kopiske et al. (2017) investigated this discrepancy in the framework of motor adaptation. One of their secondary considerations was whether there was an effect of having multiple target sizes, as the studies which showed a decrease in the illusion effect used fewer objects than the studies which showed a constant illusion effect. They found a decreasing illusion effect with repeated trials, which could be explained by an error-correction model of sensorimotor adaptation, but importantly, found no effect of presenting single vs. multiple target sizes. This finding, taken together with our results that grasping movements did not become highly stereotyped over time, suggests that using a single target size did not hinder our ability to observe an effect of ensemble perception on grasping should it exist.

A second possible limitation of our study stems from the fact that after collecting data from 10 participants, we modified the experimental procedure by correcting the starting position of the right index finger and thumb from separated in the original procedure to touching in the modified procedure and recorded the haptic feedback portion of the manual estimation trials for further analysis (see “Modifications to the Experimental Procedure” section in the Materials and Methods section for more details). Notably, these changes were specific to the grasping task, as they did not influence the perceptual results. Nevertheless, we showed that maximum grip aperture for the grasping task did not differ between the original and modified experimental procedures whatsoever. We did, however, find a subtle *overall* CCW shift in grip orientation. We attributed this overall shift in grip orientation to the change in starting posture with the modified experimental procedure, where the index finger and thumb were pinched together. Importantly, this change did not interact with any of the unique conditions, indicating that it was independent of the any of the effects of our experimental manipulations and can thus be reasonably considered moot.

### The Effects of Obstacle Avoidance and Perceptual Processing on Grasping Movements

To examine the influence of obstacle avoidance on our grasping data, we compared the baseline grasping trials (i.e., grasping the central target presented without a surrounding ensemble) to the main grasping and haptic feedback trials. We observed that MGA was larger in the baseline grasping task compared to when the target was embedded in the ensemble display (i.e., for both the main and haptic feedback grasping trials). These findings are in-line with what one would predict if obstacle avoidance mechanisms were operating on grip aperture (e.g., Saling et al., 1998; de Grave et al., 2005; Chen et al., 2015). Additionally, we observed that grip orientation was more CW in the baseline grasping task relative to the main grasping and haptic feedback grasping tasks, which may have been induced by the configuration of flanking objects (de Grave et al., 2005). Although the surrounding ensemble objects appear to induce general obstacle avoidance effects, there were no effects of variations in average size or orientation on GO in the main grasping task. Thus, it seems reasonable to conclude that the influence of the ensemble on MGA and GO, relative to no ensemble whatsoever, was general and independent of the mean ensemble size and orientation. Furthermore, obstacle avoidance is a natural component of prehension and while it is mainly controlled by dorsal-stream mechanisms, it is not completely isolated from ventral-stream processing (McIntosh et al., 2004; Schindler et al., 2004; Rice et al., 2006, 2008; Hesse et al., 2012), making it a prime target to further explore the interdependence of the two streams.

### Haptic Feedback From 2D vs. 3D Objects

In an ensemble adaptation paradigm by Corbett and Song (2014), perceptual biases were observed during the early but not late stages of grasping. In our study, we did not observe any influence of perceptual processing on grasping movements. One reason for this discrepancy is likely explained by the absence of haptic feedback from a 3D target in Corbett and Song (2014). Haptic feedback is an important aspect of grasping and when absent can shift visuomotor behavior to being governed more by ventral-stream mechanisms such as in pantomimed grasping (e.g., Bingham et al., 2007; Schenk, 2012; Fukui and Inui, 2013; Whitwell et al., 2014, 2015). Corbett and Song acknowledged this issue and asked participants to touch their fingers to the monitor to receive some visual and haptic feedback when “grasping”; however, the haptic feedback was not veridical owing to the 2D stimulus. As discussed in the Introduction section, tactile feedback from a 2D object may not be enough to restrict the visuomotor operations that specify the kinematic parameters of the grasp to those that are typical of natural grasping (Holmes and Heath, 2013; Freud and Ganel, 2015; Ozana and Ganel, 2019). We used 3D objects in our study (as did Hamidi et al., 2021), providing veridical haptic feedback, and grasping was observed to be refractory to perceptual biases.

### Future Directions

Although we observed no effects of mean ensemble size and orientation on grasping, it is possible that the present study may not have sufficiently induced a need for the visuomotor system to utilize ensemble size or orientation as the task could have been completed by simply focusing on the target object’s size. Although extraction of multiple ensemble characteristics within a single ensemble seems to occur automatically and in parallel (Chong and Treisman, 2005; Attarha and Moore, 2015; Yörük and Boduroglu, 2020), attention to a specific feature may be necessary to optimize ensemble processing within that dimension (Emmanouil and Treisman, 2008). It is plausible that no ensemble effect was observed in the grasping task because the visual system discounted ensemble size and orientation altogether as they were not directly relevant to the task. If the task was manipulated such that ensemble perception would markedly benefit performance, there may be an observable effect of ensemble statistics on visuomotor control. Such manipulations could include the use of a speeded object grasping task (e.g., where matching hand posture to the ensemble average would allow for the fastest adoption of the necessary grasp position), or the use of higher-level ensemble displays (e.g., biasing grasp behavior using real-world tools that are often handled in specific orientations). Furthermore, as we used a limited number of ensemble configurations and a constant target size, a future study should use a wider range of stimuli both in terms of the ensemble display and target objects.

Another interesting follow up experiment would explore 2D ensemble backgrounds and a 3D target, to help reduce any obstacle-avoidance effects. Finally, utilizing an ensemble display within a more conventional visual illusion paradigm where ensemble statistics directly affect the perception of the to-be-grasped target will help to put the present series of results into context.

## Conclusion

Understanding if and how the perceptual and action systems interact would deepen our understanding of both ensemble statistical processing and visuomotor control, as well as the relation between the ventral perception stream and dorsal action stream (Milner and Goodale, 2006). Practical applications of a system that uses ensemble-like processing could be in computer vision where a balance between perceptual constancy and outlier detection could guide algorithms which are robust to failure yet sensitive to unexpected conditions (i.e., those employed in self-driving vehicles). Given the benefits of ensemble perception (e.g., the ability to circumvent the capacity limitation in visual attention and visual working memory; Cohen et al., 2016), future research employing more complex paradigms will have to determine whether this mechanism could allow the visuomotor system to change focus or ignore irrelevant factors by perceiving the gist of our surroundings. As for the results of the present grasping paradigm, the physical constraints of interacting with our environment dictate that only visual information immediately relevant to motor behavior is considered by the visuomotor system.

## Data Availability Statement

The raw data supporting the conclusions of this article will be made available by the authors, without undue reservation.

## Ethics Statement

The studies involving human participants were reviewed and approved by University of Toronto Social Sciences, Humanities, and Education Research Ethics Board. The participants provided their written informed consent to participate in this study.

## Author Contributions

RW, JC, MN, and AFa contributed to the conception and design of the study. JC, MN, LG, and AFr contributed to the set-up of the experimental materials and advised the data collection and statistical analysis process. AFa generated the experimental materials, performed the data collection and statistical analysis, and wrote the first draft of the manuscript. All authors contributed to manuscript revision, read, and approved the submitted version.

### Conflict of Interest

The authors declare that the research was conducted in the absence of any commercial or financial relationships that could be construed as a potential conflict of interest.

## Footnotes

^1^https://www.letswave.org/
