# Supplementary material for: Grasping of Real-World Objects Is Not Biased by Ensemble Perception
Source: Front Psychol. 2021 Apr 12;12:597691. doi: 10.3389/fpsyg.2021.597691 (PMC8071954; doi:10.3389/fpsyg.2021.597691)
Supplement: Supplementary file 1 [file Data_Sheet_1.docx]

Supplementary Material

# Variability of Grasping Movements – Square root transformed data

# Methods

As grasps were directed to a single target that had a constant shape and size, it was possible that the grasping movement became stereotyped with repetition over the experimental session. In order to examine whether this occurred, we conducted a post-hoc analysis of the standard deviation of maximum grip aperture (MGA) and grip orientation (GO) across the grasping session (calculated for each participant; split into one bin of 10 practice trials, one bin of 10 initial baseline trials, 4 bins of 20 grasping trials each, and one bin of 10 final baseline trials). Standard deviation of MGA and GO were modelled separately as a function of trial bin, with the first bin (practice trials) as the reference level. The ICC for both models suggested that standard deviation of MGA (*ICC* = .39, *N* = 1446, *α* = .05, *p* < .00001) and GO (*ICC* = .59, *N* = 1446, *α* = .05, *p* < .00001) was clustered within participants. All models included a random intercept for participant, and the dependent variable was modelled as a function of bin. Since the data for MGA and GO had slightly non-normally distributed residuals, we re-ran the analysis on square root transformed data. The results for GO did not differ from the original, and, while the transformed vs. non-transformed results were slightly different for MGA, the overall result was the same. That is, there was no decreasing trend of variability in grasping movements over time compared with the practice trials. Here, we report the results using the transformed data for both GO and MGA (note that summary statistics are back transformed for ease of interpretation).

# Results

Because grasping movements might have become stereotyped with repetition, thereby obscuring any influence of the ensembles, we tested whether variability declined over time, by comparing variability in each grasping bin to that observed in the practice bin. However, we observed no such trend (in fact, the data trended in the opposite direction, with some bins showing significantly greater variability compared with the practice trials). There was an overall effect of bin on the standard deviation of MGA (*F*(6, 1425.28) = 25.99, *p* < .0001, *R^2^* = .10) and GO (*F*(6, 1425.13) = 13.82, *p* < .0001, *R^2^* = .06). The standard deviation of MGA was smaller in the practice trials (*M* = 2.25, *SE* = .002, *95% CI* = [2.02, 2.53]) compared to the 1^st^ and 4^th^ quartile of the grasping trials (*M_Grasp bin1_* = 2.37, *SE* = .001, *95% CI* = [2.13, 2.62]; *b* = .04, *SE* = .02, *t*(1426) = 2.18, *p* = .030; *M_Grasp bin 4_* = 2.53, *SE* = .001, *95% CI* = [2.28, 2.79]; *b* = .08, *SE* = .02, *t*(1425) = 4.55, *p* < .0001) and the final baseline trials (*M_Final_* _baseline_ = 2.96, *SE* = .002, *95% CI* = [2.69, 3.24]; *b* = .22, *SE* = .02, *t*(1425) = 10, *p* < .0001; see Supplementary Figure 1A). The standard deviation of GO was smaller in the practice trials (*M* = 3.92, *SE* = .01, *95% CI* = [3.03, 4.93]) compared with all other baseline and grasping bins (all *ts >*4.91 all *ps* < .0001; see Supplementary Figure 1B).

# Figure

**
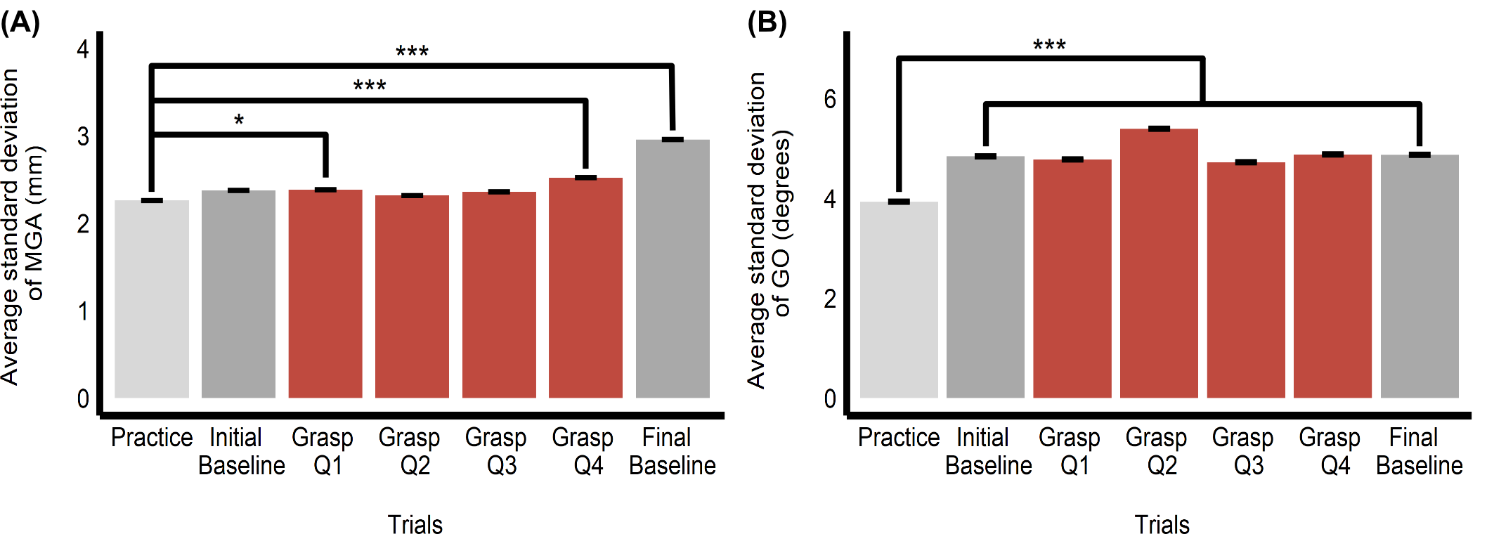
**

**Supplementary Figure 1. Variability of grasping movements.** Back transformed (squared) estimated marginal means of average standard deviation of (A) maximum grip aperture and (B) grip orientation across the grasping trials (split into one bin of 10 practice trials, one bin of 10 initial baseline trials, 4 bins of 20 grasping trials each (Q1–Q4), and one bin of 10 final baseline trials). Comparisons are made between practice trials and all other bins. * *p* < .05, *** *p* < .0001; error bars depict SEM; MGA = maximum grip aperture; GO = grip orientation.
